# Enhanced UV Emission From Silver/ZnO And Gold/ZnO Core-Shell Nanoparticles: Photoluminescence, Radioluminescence, And Optically Stimulated Luminescence

**DOI:** 10.1038/srep14004

**Published:** 2015-09-14

**Authors:** E. J. Guidelli, O. Baffa, D. R. Clarke

**Affiliations:** 1 School of Engineering and Applied Sciences, Harvard University, Cambridge, MA 02138; 2 Departamento de Física - FFCLRP- Universidade de São Paulo, Brazil

## Abstract

The optical properties of core-shell nanoparticles consisting of a ZnO shell grown on Ag and Au nanoparticle cores by a solution method have been investigated. Both the ZnO/Ag and ZnO/Au particles exhibit strongly enhanced near-band-edge UV emission from the ZnO when excited at 325 nm. Furthermore, the UV intensity increases with the metal nanoparticle concentration, with 60-fold and 17-fold enhancements for the ZnO/Ag and ZnO/Au, core-shell nanoparticles respectively. Accompanying the increase in UV emission, there is a corresponding decrease in the broad band defect emission with nanoparticle concentration. Nonetheless, the broad band luminescence increases with laser power. The results are consistent with enhanced exciton emission in the ZnO shells due to coupling with surface plasmon resonance of the metal nanoparticles. Luminescence measurements during and after exposure to X-rays also exhibit enhanced UV luminescence. These observations suggest that metal nanoparticles may be suitable for enhancing optical detection of ionizing radiation.

Noble metal nanoparticles, such as gold and silver, have distinctive properties useful for technological applications in physics, chemistry, biology, and medicine. For instance, metal nanoparticles have been employed in radiation therapy treatments of cancer^1^,^2^, as X-Ray contrast agents^3^,^4^ and drug delivery systems^5^, as well as in sensors and detectors^6^,^7^,^8^,^9^,^10^. In addition to their potential applications, noble metal nanoparticles are also of interest on account of the ability to tune their surface plasmon absorption band by changing their size. The plasmon absorption band is a result of the interaction between the electric field of light and the collective oscillation of the conduction electrons at the nanoparticles surfaces^11^. Under such resonance conditions, metal nanoparticles can have an optical cross-section (extinction coefficient) much larger than their physical cross section. For instance, the optical cross-sections of metal nanoparticles under resonant conditions can increase by factors up to 10^5^ 12,13,14.

The interaction of plasmons with the surrounding medium leads to new optical features. For instance, energy and electron transfer from plasmon resonant metal surfaces to adjoining semiconductors can dramatically alter their optical properties, making metal-semiconductor nanocomposites a new class of multifunctional optical components^15^, specially nanoparticles with a core/shell configuration. In fact, metal-semiconductor core/shell nanoparticles have been proposed for a variety of applications such as biomedical and pharmaceutical analysis, catalysis, and electronics^16^,^17^,^18^,^19^,^20^.

In this work, we investigate the optical properties, including after ionizing irradiation, of core-shell nanoparticles consisting of ZnO deposited onto silver and gold nanoparticles. ZnO has been chosen as the shell material because its near-band-edge and defect-related ZnO emissions are close in energy to the plasmon resonances of Ag and Au, raising the possibility of luminescence enhancements. In addition to its potential applications in many developing technologies such as sensing^21^,^22^, light harvesting^23^, catalysis^24^, drug activation for radiation therapy of cancer^25^, ZnO has also been utilized in the detection of ionizing radiation, for instance as a radio-luminescent, thermo-luminescent, and optically stimulated luminescence detector^16^,^20^,^26^,^27^,^28^. Radioluminescence, the emission of light during exposure to ionizing radiation, such as X-Rays, is an instantaneous process and does not specifically involve electron trapping or charge storage. In contrast, optically stimulated luminescence (OSL) is similar in principle to the classical thermoluminescence (TL) process, and is based on the stimulated emission of light, rather than thermally as in the case of TL^29^, of a previously irradiated material. Electrons trapped in the band gap as a result of exposure to the ionizing radiation, are then excited by optical stimulation (usually green or blue light), producing emission in the ultra-violet region^30^. The significance of OSL is that light having energy greater than the energy of the excitation can be detected which is in marked contrast to other, non-dosimetric luminescence processes, such as photoluminescence^30^.

Following a description of the synthesis and microstructure of our ZnO particles containing silver and gold nanoparticle cores, the effect of laser power and volume fraction of nanoparticles on the enhanced emission of light under UV excitation is described. The effect of volume fraction of core-shell nanoparticles on the radio-luminescence and optically stimulated luminescence are then quantified. The observations are discussed in terms of enhancement of the principal features of the optical spectra of ZnO by surface plasmons in the nanoparticle cores. Finally, the prospects for utilizing these spectral enhancements in OSL radiation detectors are discussed.

## Materials and Methods

### Synthesis of the Silver and Gold Nanoparticles

Silver and gold nanoparticles were synthesized by a microwave assisted polyol-method based on a synthetic route^31^, with some modifications, as follows: They were synthesized from mixtures of 45 mg of silver nitrate (AgNO_3_) or 49 mg of Gold(III) chloride hydrate (HAuCl_4_), and 110 mg of polyvinylpyrrolidone (PVP) dissolved in 100 mL of ethylene-glycol (EG), resulting in silver and gold final concentrations of 2.66 mmol.L^−1^ and 1.44 mmol.L^−1^. All chemicals were reagent grade, purchased from Sigma-Aldrich. The solutions were microwaved (1200 W) for 120 seconds, stirring every 30 seconds, yielding yellow and red solutions of silver and gold nanoparticles, respectively, in colloidal suspension. UV-Vis spectroscopy revealed a plasmon resonance band peaking at 420 nm and 520 nm, for silver and gold nanoparticles, respectively, confirming the formation of metal nanoparticles. TEM images (not shown here) revealed spherical particles with average sizes around 30 nm for silver and 80 nm for gold.

### Preparation of ZnO/Ag and ZnO/Au shell-core Nanoparticles

A three step process was used to produce the shell-core nanoparticles on a ZnO coated substrate. In the first step, clean glass substrates were dipped into a 0.01 mol.L^−1^ zinc acetate dihydrate Zn(CH_3_COO)_2_ · 2H_2_O aqueous precursor solution for 5 minutes as reported elsewhere^32^. They were then rinsed with Milli-Q^TM^ water and annealed at 500 °C for 10 minutes. This procedure was then repeated three times to form a continuous coverage of the glass substrate.

In the second step, the ZnO coated glass substrates were placed in the bottom of a large glass bottle containing 40 mL of a 0.5 mol.L^−1^ zinc nitrate hexahydrate (Zn(NO_3_)_2_.6H_2_O) aqueous solution with 6% vol. of ammonium hydroxide (NH_4_OH), at room temperature. In the third step, solutions of the metal nanoparticles were added and, after sealing, the bottles were kept at 100 °C overnight. Samples were prepared from 0.1, 0.5, 1, 2 4, and 8 mL of silver/gold nanoparticles suspension which led to final concentrations of 0.006, 0.032, 0.065, 0.13, 0.24, and 0.44 mmol.L^−1^ of silver; and 0.003, 0.018, 0.035, 0.07, 0.13, and 0.24 mmol.L^−1^ of gold. The final volume fraction of the metal nanoparticles in the films could not be established with any certainty so forthwith the samples will be referred to by the volume concentration of the nanoparticles used to make the films. Initially, the solutions containing silver and gold nanoparticles were yellow and red, respectively. After the step in which the ZnO was precipitated, the solution became transparent, indicating that the noble metal nanoparticles became engulfed by the ZnO phase. Subsequently, each of the samples was annealed at 300 °C for 2 h to eliminate any residual water. For reference purposes, a control sample of ZnO without any silver/gold nanoparticles was also prepared.

### Sample Characterization

Each of the samples was characterized by X-ray diffraction and scanning electron microscopy as well as optically. The X-ray diffraction (XRD) patterns were acquired using a Bruker D8-Discover diffractometer, over the range 30° ≤ 2θ ≤ 65°, with an increment of 0.02°. The CuKα emission line (1.541 Å, 40 kV, 40 mA) coupled to a germanium monochromator was used. The morphology of the particles was evaluated using a field emission scanning electron microscopy Zeiss - FESEM-Ultra Plus equipped with electron backscattering (ESB) detector and a charge compensation system to reduce charging making it unnecessary to coat the samples. UV-Vis spectroscopy was performed with a Hitachi U-4001 UV-VIS spectrophotometer equipped with an integrating sphere detector. Photoluminescence (PL) spectroscopy was performed using a LabRAM Aramis spectrometer (Horiba – Jobin Yvon, Edison, NJ), using 532 nm and 325 laser excitations, respectively. Unless otherwise stated, all the photoluminescence measurements were made using UV excitation with a 1.1 mm diameter optical aperture under the same optical conditions. The incident laser power was varied using filters with different optical densities. In separate experiments, using a Tsunami 3950 Spectra Physics titanium–sapphire laser, pumped by a solid state Millenia X Spectra Physics laser, photoluminescence decays were measured using a time-correlated single-photon counting (TCSP) method. The repetition rate of the 5 ps pulses was set to 8 MHz using the 3980 Spectra Physics pulse picker. Excitation was at 308 nm and the emission was collected at 385 nm. Emitted photons were detected by a refrigerated Hamamatsu R3809U microchannel plate photomultiplier. Software provided by Edinburgh Instruments was used to analyze the decay curves. The quality of the multi-exponential decay fitting was judged by inspection of the plots of weighted residuals and by statistical parameters such as reduced chi-square.

Radioluminescence measurements were performed under X-Ray irradiation from a X-ray tube (Magnun - Moxtek, USA), operating at 48 kVp and 0.2 mA. The emitted light was collected and analyzed using a fiber-optic spectrometer. The X-ray dose rate at the sample position is around 9 Gy/min. In addition, samples were also irradiated in air with a 500 Gy dose and using the same X-Ray tube. Optically stimulated luminescence was acquired using an OSL reader developed by the Laboratory of Dosimetry and Nuclear Instrumentation, Universidade Federal de Pernambuco. A blue LED with maximum emission at 470 nm is used to excite the samples after the X-ray exposure and the luminescence detected with a photomultiplier and a Hoya U340 notch filter having a transmittance window of 270–370 nm. OSL signals were recorded from 5 different samples of each nanoparticle concentration.

## Results

**Figure 1:**
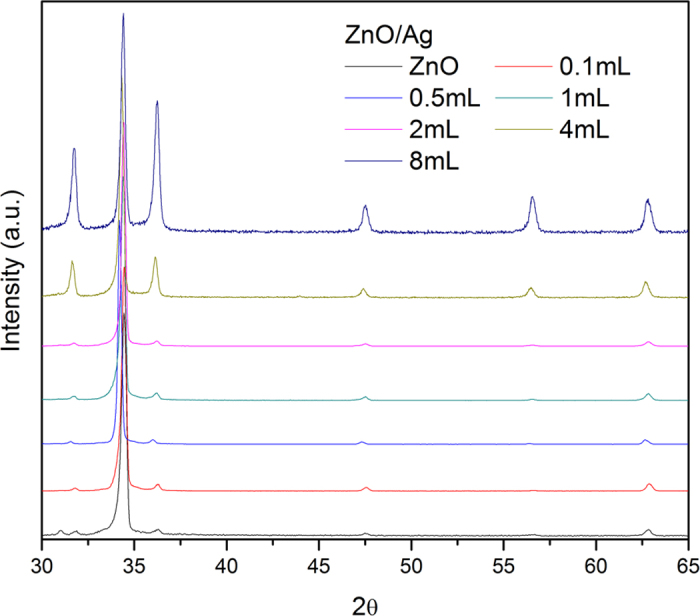
XRD patterns for ZnO/Ag shell-core nanoparticles. The original ZnO is highly textured but with increasing concentration of Ag nanoparticles, the film becomes progressively more polycrystalline. The spectra are normalized to the height of the (002) peak of ZnO.

**Figure 2:**
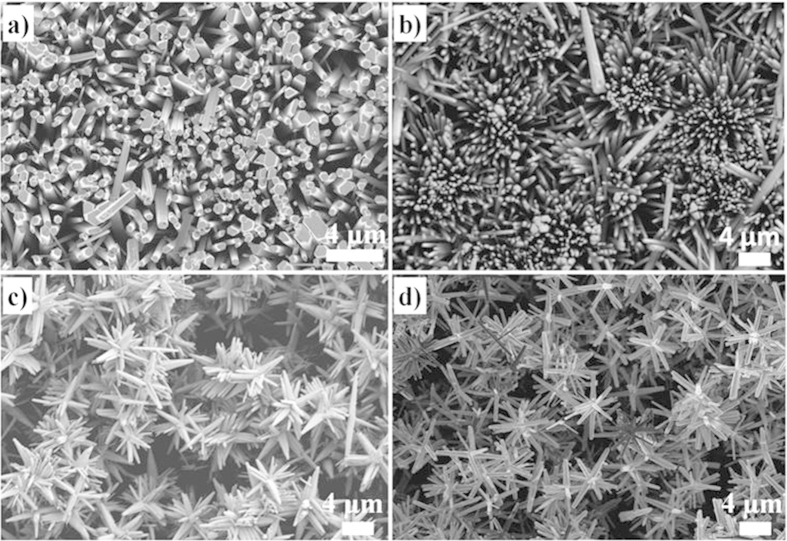
SEM images for ZnO samples without nanoparticles (a), with 2 mL_AgNP (b), 8 mL_AgNP (c), and 8 mL_AuNP (d) illustrating the change in shape of the particles. The particles in the lower two micrographs are referred to as “star” or “thistle” shaped in the text.

**Figure 3:**
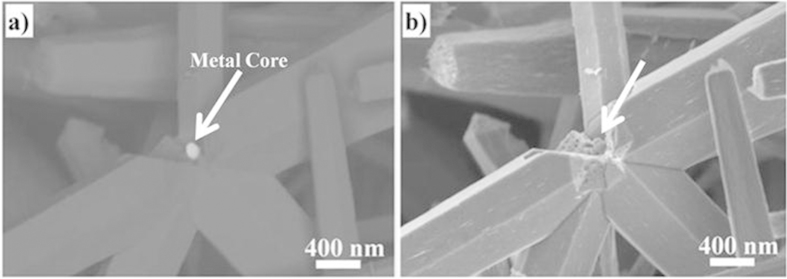
(**a**) SEM back-scatter electron image, an imaging mode that accentuates atomic number contrast, showing a gold nanoparticle (arrowed) at the core of a “star” shaped ZnO particle. (**b**) Identical region as in (**a**) but imaged using only secondary electrons to reveal the ZnO arms emanating from the gold nanoparticle.

In the absence of the metal nanoparticles, the growth of ZnO occurs preferably in the [0002] direction, suggesting the formation of a well textured c-axis wurtzite structured film or well-aligned ZnO rod particles^33^ (Fig. 1). The subsequent growth of the ZnO in the presence of the metal nanoparticles alters the ZnO film crystallography becoming progressively more polycrystalline as shown in Fig. 1. No peaks from either silver or gold were detected, presumably because of their low volume fraction. The change in growth crystallography with metal nanoparticles is confirmed by images of the films formed with different concentrations of metal nanoparticles as shown in Fig. 2. Absent any nanoparticles, reasonably well aligned ZnO nanorods grow on the glass substrate, Fig. 2(a), and this morphology is little changed with 2 mL of silver nanoparticles. However, for samples containing volumes above 4 mL of silver nanoparticles and 1 mL of gold nanoparticles, “star-like” or “thistle-like” ZnO particles were produced (Fig. 2(c,d)). The appearance of these particles is consistent with the random, polycrystalline orientation of the ZnO indicated by XRD in Fig. 1. Together, these observations suggest that the ZnO nucleates on the metal nanoparticles suspended in solution and grows thicker during the overnight hold at 100 °C. Confirmation of this is shown in the electron backscatter image of Fig. 3, which shows a metal particle at the center of one of the ZnO “stars” revealed where some of the ZnO arms have been broken off. (The substantially higher mass contrast sensitivity in the backscatter mode of operation than in the standard secondary emission mode causes the gold nanoparticle to appear much brighter than the surrounding ZnO). Moreover, no metal nanoparticles could be observed on the surface of the ZnO particles, including when observed in the electron backscattering imaging mode, indicating that all the metal nanoparticles, originally in solution, were coated by a ZnO shell.

**Figure 4:**
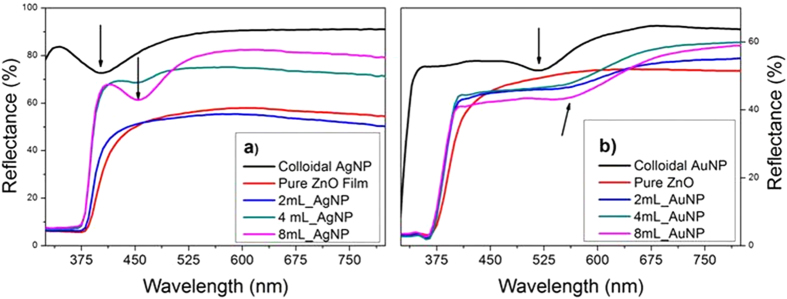
Comparison of the UV-Vis reflectance of the (a) Ag/ZnO and (b) Au/ZnO core-shell nanoparticles compared to those of the colloidal suspensions of the metal nanoparticles. The absorption band, arrowed, shifts to longer wavelengths when the nanoparticles are surrounded by the ZnO shell. This is attributed to the higher refractive index of the ZnO relative to water.

As microscopy shows that the samples are not smooth films, the optical absorption measurements were made by diffuse reflectance rather than by normal reflectance. Figure 4 shows the diffuse reflectance spectra of the ZnO samples as well as of the silver and gold nanoparticles in colloidal suspension. The plasmon bands of the colloidal silver and gold nanoparticles in solution are at 400 nm and 520 nm, respectively. The strong optical absorption from the shell-core nanoparticles with a maximum at about 360 nm is characteristic of ZnO direct band-gap transition. The distinctive plasmon absorption band of the silver nanoparticles shifted to 450 nm for the samples containing 4 and 8 mL of AgNP, suggesting that the silver particles are stable and well dispersed in the ZnO structure. The plasmon band red-shifts but does not detectably broaden, suggesting that the red-shift is caused by the change in the surrounding medium and not by the particle agglomeration. In this case the change from the nanoparticles being surrounded by EG/PVP and water to being embedded in ZnO, which has a higher refractive index^5^,^34^. The absence of an observable silver plasmon band for samples with a smaller number of silver nanoparticles, namely the 0.1 mL-AgNP, 0.5 mL-AgNP, 1 mL-AgNP, and 2 mL-AgNP, is attributed to an insufficient silver concentration combined with the high optical absorbance of the ZnO in the UV-visible range. The absorption in the 400–800 nm region is abruptly reduced for samples 4 mL-AgNP and 8 mL-AgNP compared to samples of pure ZnO, 0.1 mL-AgNP, 0.5 mL-AgNP, 1 mL-AgNP, and 2 mL-AgNP, allowing the detection of the plasmon absorption band. For the case of the ZnO/gold nanoparticles, the characteristic plasmon absorption band of gold nanoparticles is at 550 nm, detectable for gold nanoparticles volumes greater than 0.5 mL.

**Figure 5:**
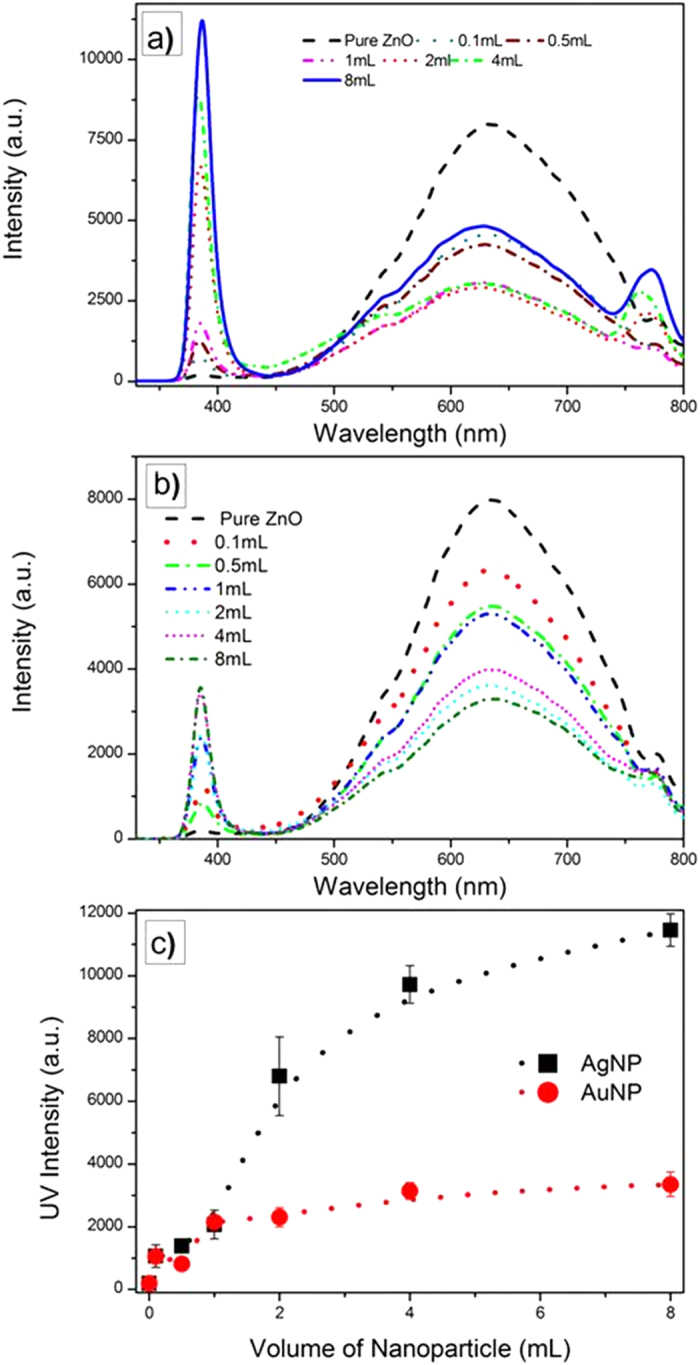
Photoluminescence spectra for the (a) ZnO/Ag and (b) ZnO/Au nanoparticles under excitation at 325 nm. (**c**) The ZnO UV-band intensity increases with increasing the metal nanoparticle concentration. Error bars represent the standard deviation for three separate measurements.

Figure 5 presents the photoluminescence spectra from both the silver and gold nanoparticle samples with ZnO shells excited at a constant laser power at 325 nm. All the spectra exhibit similar PL features, consisting of three main emission bands, between 350–450 nm, 450–750 nm, and 750–800 nm. The observations show that both silver and gold nanoparticles are found to enhance the luminescence of the UV band while simultaneously the broad band ZnO luminescence, in the range between 450–750 nm, decreases compared to that of the pure ZnO which is shown on the same plots. Furthermore, as shown in Fig. 5(c), the intensity of the ZnO UV-band is proportional to the metal nanoparticle concentration, saturating at larger volume concentrations. The UV emission band is related to the near band-edge emission from the ZnO direct band gap. The origin of the band between 750–800 nm is more controversial: Some authors have attributed it to defects in the ZnO structure while some other authors have suggested that such emission is a second-order feature of the UV emission^35^,^36^.

**Figure 6:**
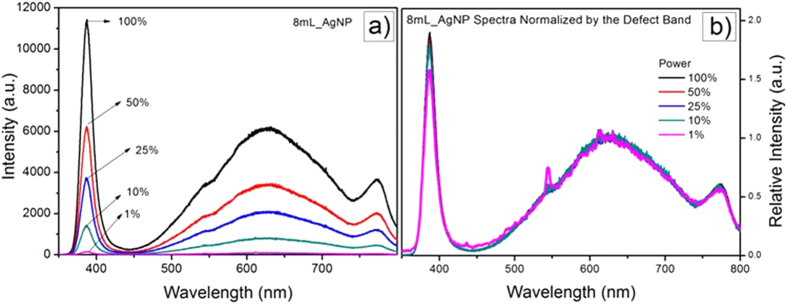
(**a**) Spectra for ZnO/Ag as a function of laser power and (**b**) the same data normalized by the defect band emission.

**Figure 7:**
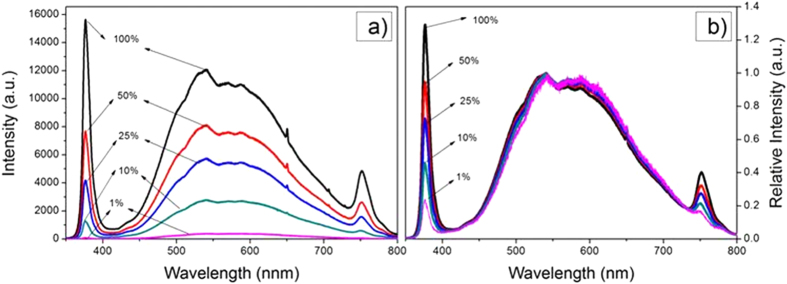
(**a**) ZnO photoluminescence (single crystal) spectra recorded at the laser powers indicated. (**b**) The same data normalized by the defect emission band. The UV band intensity, relative to the defect band, increases with the laser power, suggesting that under high electric fields, the ZnO luminescence emission occurs preferentially by the spontaneous emission from free excitons.

The dependence of the PL on the laser power at 325 nm is shown in Fig. 6 for the material containing 8 mL of silver nanoparticles. Notably, both the UV and broad band luminescence increase with laser power, exhibiting almost the same dependence as indicated in the same data but plotted in Fig. 6(b) with the spectra normalized by the maximum intensity of the broad band luminescence. For comparison purposes, the photoluminescence from a ZnO single crystal is shown in Fig. 7. As has been reported elsewhere, both the band edge luminescence and the defect band emission of single crystal ZnO increase with laser power. Significantly, though, the band-edge luminescence intensity increases much more rapidly with laser power than the broad band luminescence.

**Figure 8:**
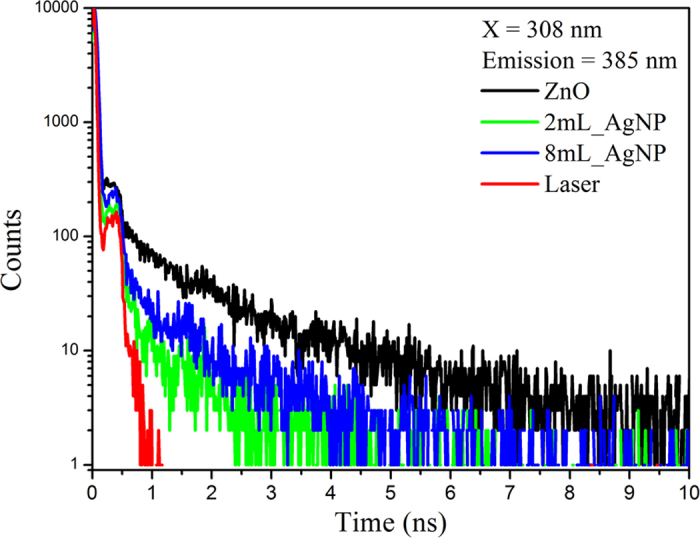
Time-Resolved Photoluminescence decays, at room temperature, under excitation at 308 nm and emission at 385 nm, of ZnO and of 2mL_AgNP and 8mL_AgNP. The decreased lifetime for the samples containing silver nanoparticles is attributed to plasmon-coupled emission mechanism.

Analysis of the luminescence decays, obtained by time resolved photoluminescence measurements, shown in Fig. 8, reveal a bi-exponential behavior for the samples of pure ZnO as well as those containing 2 mL_AgNP, and 8 mL_AgNP. The lifetimes determined are: τ_1_ = 12 ps and τ_2_ = 1.570 ns for the pure ZnO; and τ_1_ = 15 ps and τ_2_ = 1.017 ns for 2 mL_AgNP/ZnO; and τ_1_ = 19 ps and τ_2_ = 1.045 ns for 8 mL_AgNP/ZnO. According to the literature, the fastest decay component (τ_1_) is associated with non-radiative recombinations (trapping, for example), whereas the slow component (τ_2_) is attributed to the radiative lifetime of the free excitons. Therefore, with increasing silver nanoparticle concentration, there is an increase of the component associated with non-radiative transitions, τ_1,_ and a decrease of the component associated with radiative ZnO emission τ_2_. The reduction of the excitons lifetime (τ_2_) is a characteristic evidence of plasmon-coupled-emission, consisting of an energy transfer process from the ZnO excitons to the AgNP plasmons^12^,^37^,^38^.

**Figure 9:**
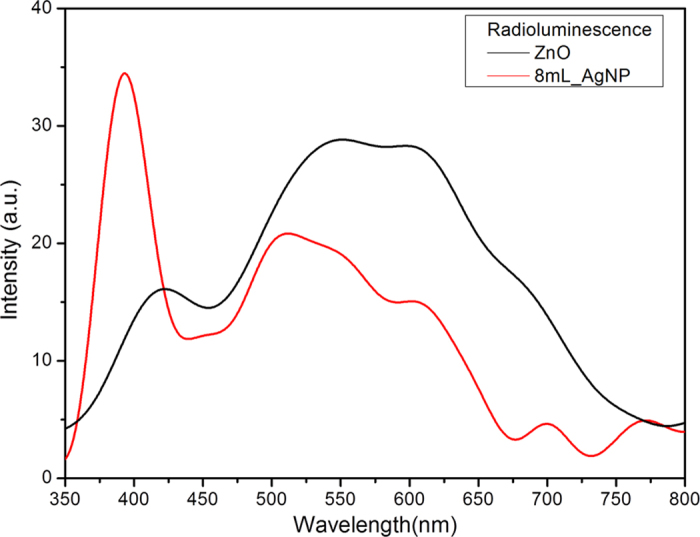
Luminescence spectra of ZnO and 8 mL_AgNP measured while being irradiated by X-Ray excitation (radioluminescence). The enhanced UV emission is consistent with a plasmon-coupled-emission mechanism in the presence of the silver nanoparticles.

**Figure 10:**
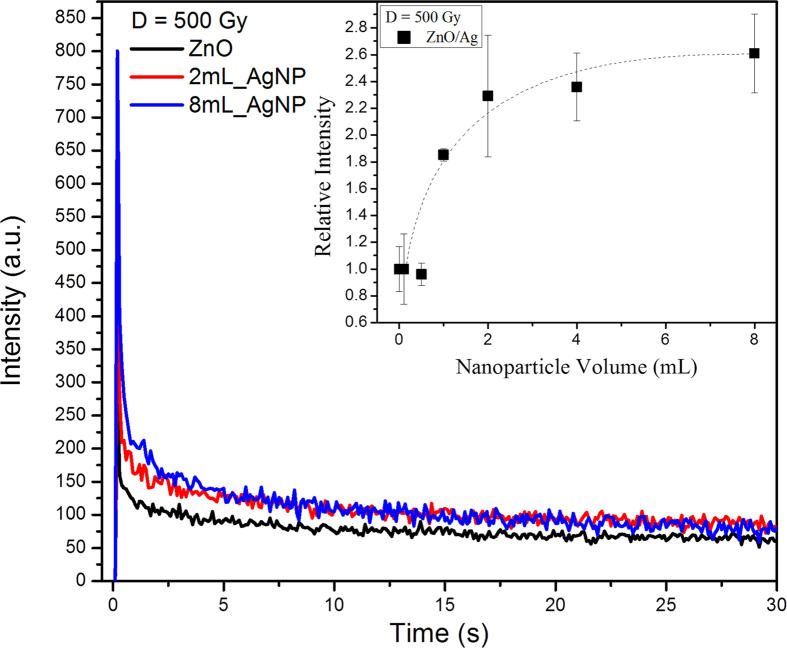
Optically stimulated luminescence collected over the range 270–370 nm from pure ZnO and ZnO films containing silver nanoparticles after exposure to an X-ray dose of 500 Gy. The luminescence was stimulated at 470 nm. The inset depicts the integrated intensity as a function of the silver nanoparticles volume. The error bars represent the experimental standard deviation for five samples.

**Figure 11:**
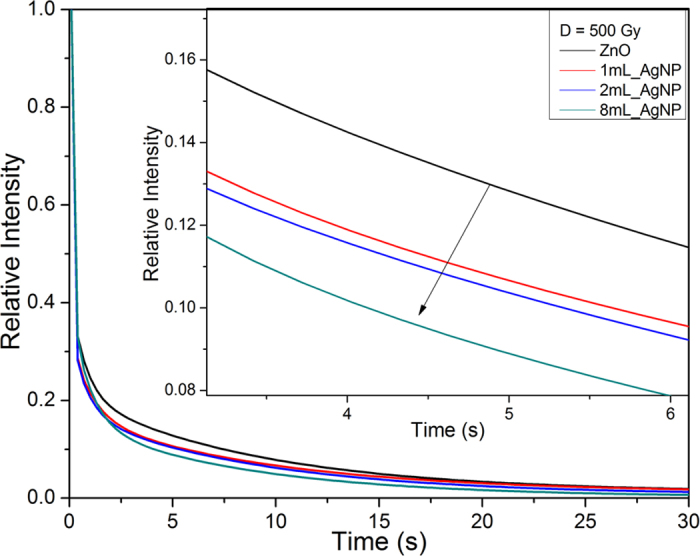
OSL curves normalized by the initial intensity. Faster decay of the OSL curve is observed with increasing silver nanoparticle concentration, suggesting a faster emptying of traps in the ZnO.

Figure 9 compares the luminescence spectra of ZnO and the 8 mL_AgNP materials under X-Ray excitation (radioluminescence). As with the observations recorded with direct laser excitation (Fig. 5), there is both an increase in the UV band as well as a reduction of the defect band intensity for the samples containing silver nanoparticles. In addition, optically stimulated luminescence (OSL) measurements indicate that there is an increase with the volume of Ag nanoparticles (Fig. 10). When the OSL measurements were repeated, there was no further OSL intensity suggesting that the trapped charge was released during the first measurement. Furthermore, the OSL decay time is progressively shorter with increasing number of nanoparticles (Fig. 11), suggesting that trapped charges from the ZnO defect states produced by the ionizing X-ray radiation empty at a higher rate.

## Discussion

Our photoluminescence and radioluminescence measurements demonstrate that Ag/ZnO and Au/ZnO core-shell nanoparticles exhibit enhanced UV luminescence compared to the as prepared pure ZnO and to the as prepared pure ZnO. The enhancement is linearly proportional to the laser power under photoluminescence conditions and also increases with volume of nanoparticles before approaching an apparent saturation value. Furthermore, the radioluminescence measurements clearly showed a marked UV luminescence enhancement relative to ZnO without nanoparticles. These measurements, together with the increase in the optically stimulated luminescence with the number of nanoparticles after X-ray irradiation, suggest that the use of the Ag/ZnO nanoparticles may hold promise for more sensitive radiation detectors.

The origin of the UV luminescence, typically in the range of 360 to 420 nm, from ZnO is generally attributed to the photo-stimulated creation of excitons having energies just below the ZnO band-edge and their subsequent recombination. Therefore, the explanation for the observed enhancements must be associated with changes in the probability of creating excitons and their recombination lifetimes. In the case of pure ZnO, the enhancement of the UV luminescence with laser power^39^ is due to the increased number of excitons created as well as an increase in excitonic recombination efficiency in the electric field of the illuminating laser. In the presence of the Ag and Au nanoparticles, the local field is also greatly enhanced by the surface plasmon resonance. As has been analyzed elsewhere^14^, under plasmon resonance conditions an evanescent electric field is created at the surface of the nanoparticles. This field penetrates into the surrounding ZnO and augments the electric field of the illuminating laser. Models for the depth of penetration of the evanescent wave into the ZnO shell, assuming it is thick compared to the nanoparticle radius, show that the strength of the field falls off in proportion to the sixth power of the radius^40^ if the particles are spherical. Based on the assumption that the volume of the ZnO in which the evanescent wave couples to the excitons is also proportional to the number of nanoparticles, the increase in the UV intensity should increase with the volume of nanoparticles. The measurements in Fig. 5 are consistent with this for small volumes until saturating at larger volumes. As the films are essentially porous and granular, the saturation is attributed to self-absorption as the stimulated UV intensity propagates. Direct support for the role of the nanoparticles in increasing the exciton recombination efficiency comes from the time-resolved photoluminescence measurements (Fig. 8) that indicate a decrease in exciton lifetime in the presence of the nanoparticles.

One of the unexpected findings was that the photo-stimulated broad band luminescence decreased with increasing volume of nanoparticles while the UV luminescence increased (Fig. 5). The data also shows that for the coating containing the highest volume fraction of Ag core-shell particles, the UV and broad-band luminescence both increase together with increasing laser excitation power (Fig. 6). This is in marked contrast with the power dependence of the luminescence from single crystal ZnO reported previously^39^,^41^, and shown in Fig. 7, where both the broad band and band-edge luminescence increase with laser power but the UV luminescence increases at a higher rate. (The data in Figs 5, 6 and 7 were all recorded under similar acquisition conditions, so the intensities are comparable). According to the literature, the broad band luminescence, extending over the approximate range of 450–750 nm, is an overlap of several peaks related to defects in the ZnO structure such as zinc and oxygen vacancies and/or interstitials^35^,^36^,^42^. The emission process is generally understood to be the recombination of a hole with an F center (electron associated with an oxygen vacancy)^42^. Thus, the presence of the nanoparticles appears to quench the recombination process.

A similar reduction in the broad band luminescence with laser power has been noted previously^43^,^44^,^45^,^46^ but not quantified. The effect of nanoparticle concentration on the luminescence was also not reported. In these earlier reports, the decrease in broad band luminescence was attributed to electron transfer across the metal/ZnO interface, from ZnO defect states to the metal surface. Specifically, the plasmon resonance causes a fraction of the trapped electrons to be pumped into the ZnO conduction band, thereby quenching the defect emission in the surrounding ZnO. While such charge transfer may be possible in other configurations, this does not seem applicable in our work as there is no possibility of a corresponding flow of electron holes to maintain electrical neutrality as the particles are electrically isolated. Furthermore, as the arms of the ZnO “stars” are much larger (up to several microns) than the noble metal nanoparticles, it is difficult to see how the defect emission over such large distances are affected by local charge transfer. Nevertheless, plasmon/fluorescent center interactions have been reported to occur over distances of several hundred nanometers^12^,^47^. As we also observe a reduction of the defect band emission under X-Ray excitation, i.e., with photons that do not match the plasmon resonance band of the Ag nanoparticles, we believe it is more likely that this quenching is probably associated with the absorption of photons by the plasmon band.

The radioluminescence and OSL results are of particular interest because of their relevance to radiation monitoring. Many radiation detectors are based on being able to read out defects created by ionizing radiation, which in the case of ZnO, are point defects. This is typically done post-irradiation by thermoluminescence, optically stimulated luminescence, and electron spin resonance spectroscopy. Our OSL observations, which are recorded over the range of 270–370 nm, indicate that in the presence of the nanoparticles the defects produced by X-ray irradiation, can also be read out from the enhanced UV luminescence. This, in turn, implies that there is a more efficient conversion of trapped electrons into optically stimulated luminescence in the UV region. The evidence for this is that the OSL intensity is not only significantly higher for samples containing silver nanoparticles but also, as shown in the inset of Fig. 10, the intensity has a similar dependence on the nanoparticle volume as does the PL intensity shown in Fig. 5(c). In addition, the more rapid decrease of the OSL intensity with stimulation time with increasing silver nanoparticle volume indicates a faster de-trapping of charges (Fig. 11). Together, the increase in OSL, the faster decay as well as the dependence on the nanoparticles at the core of the ZnO shells suggests that this is a result of surface plasmon coupling.

Further work is needed to clarify the detailed mechanisms by which the surface plasmons in the silver nanoparticle cores couple to the defect states in the surrounding ZnO produced by ionizing radiation. However, these observations clearly show that the use of nanoparticles consisting of a noble metal core and a luminescent shell may be a useful direction in the development of detectors for ionizing radiation.

### Summary and Conclusions

ZnO particles with cores of silver and gold nanoparticles have been successfully synthesized. During growth, the ZnO nucleates on the surface of the pre-existent silver and gold nanoparticles, engulfing them and subsequently growing into a “star-shaped” morphology. The shell-core particles exhibit strongly increased ZnO near-band-edge (UV) emission centered at 385 nm and reduced defect band intensity relative to single crystal ZnO. Increasing the number of nanoparticles produces similar enhancement of the near-band-edge emission intensity as is observed to occur when increasing the laser power when exciting ZnO crystals. Enhancement of the near-band-edge UV luminescence from the nanoparticles also occurs when illuminated with 40 kV X-rays. The enhanced luminescence as well as the decreased free exciton lifetime, suggest a plasmon-coupled-emission mechanism; whereas the faster de-trapping of charges from ZnO defects indicates that higher excitation rates due to increased local electric fields close to the metal nanoparticles under plasmon resonance conditions also takes place. Therefore, the plasmon properties of metal nanoparticles lead to a more efficient conversion of trapped electron into optically stimulated luminescence. This suggests that plasmon-coupled luminescence can be employed for the development of improved radiation detectors.

## Additional Information

**How to cite this article**: Guidelli, E. J. *et al.* Enhanced UV Emission From Silver/ZnO And Gold/ZnO Core-Shell Nanoparticles: Photoluminescence, Radioluminescence, And Optically Stimulated Luminescence. *Sci. Rep.* **5**, 14004; doi: 10.1038/srep14004 (2015).
